# Intracranial hemorrhage in adult patients with hematological malignancies

**DOI:** 10.1186/1741-7015-10-97

**Published:** 2012-08-29

**Authors:** Chien-Yuan Chen, Chan-Hwei Tai, Aristine Cheng, Hung-Chang Wu, Woei Tsay, Jia-Hau Liu, Pey-Ying Chen, Shang-Yi Huang, Ming Yao, Jih-Luh Tang, Hwei-Fang Tien

**Affiliations:** 1 Department of Internal Medicine, National Taiwan University Hospital, Taipei, Taiwan; 2 Department of Neurology, National Taiwan University Hospital, Taipei, Taiwan; 3 Department of Internal Medicine, Division of Hematology and Oncology, Chi-Mei Hospital, Tainan, Taiwan; 4 Tai-Cheng Stem Cell Therapy Center, National Taiwan University Hospital, Taipei, Taiwan; 5 Department of Nursing, National Taiwan University Hospital, Taipei, Taiwan

**Keywords:** central nervous system (CNS) involvement, cerebral hemorrhage, hematological malignancy, prognosis, neuroimage

## Abstract

****Background**:**

Clinical characteristics and outcomes of intracranial hemorrhage (ICH) among adult patients with various hematological malignancies are limited.

****Methods**:**

A total of 2,574 adult patients diagnosed with hematological malignancies admitted to a single university hospital were enrolled into this study between 2001 and 2010. The clinical characteristics, image reports and outcomes were retrospectively analyzed.

**Results:**

A total of 72 patients (48 men and 24 women) with a median age of 56 (range 18 to 86) had an ICH. The overall ICH incidence was 2.8% among adult patients with hematological malignancies. The incidence of ICH was higher in acute myeloid leukemia (AML) patients than in patients with other hematological malignancies (6.3% vs 1.1%, *P *= 0.001). ICH was more common among patients with central nervous system (CNS) involvement of lymphoma than among patients with CNS involved acute leukemia (*P *<0.001). Sites of ICH occurrence included the cerebral cortex (60 patients, 83%), basal ganglia (13 patients, 18%), cerebellum (10 patients, 14%), and brainstem (5 patients, 7%). A total of 33 patients (46%) had multifocal hemorrhages. In all, 56 patients (77%) had intraparenchymal hemorrhage, 22 patients (31%) had subdural hemorrhage, 15 patients (21%) had subarachnoid hemorrhage (SAH), and 3 patients (4%) had epidural hemorrhage. A total of 22 patients had 2 or more types of ICH. In all, 46 (64%) patients died of ICH within 30 days of diagnosis, irrespective of the type of hematological malignancy. Multivariate analysis revealed three independent prognostic factors: prolonged prothrombin time (*P *= 0.008), SAH (*P *= 0.021), and multifocal cerebral hemorrhage (*P *= 0.026).

**Conclusions:**

The incidence of ICH in patients with AML is higher than patients with other hematological malignancies. But in those with intracranial malignant disease, patients with CNS involved lymphoma were more prone to ICH than patients with CNS involved acute leukemia. Mortality was similar regardless of the type of hematological malignancy. Neuroimaging studies of the location and type of ICH could assist with prognosis prediction for patients with hematological malignancies.

## Background

In adult patients with hematological malignancies, infection is the most common complication. Intracranial hemorrhage (ICH) is the second most common complication, and is associated with high morbidity and mortality [1-4]. Several comprehensive reviews have highlighted the following risk factors for ICH in cancer patients: hypertension, vessel wall abnormality, invasion or compression of vessels from a tumor in or adjacent to the brain, low platelet count or platelet dysfunction, coagulation factor deficiency, disseminated intravascular coagulation (DIC), sepsis, and hyperleukocytosis [1,5-8]. Hematological malignancies comprise a diverse group of neoplasms, and may directly or indirectly lead to neurological complications and ICH [9,10]. In recent decades, prophylactic platelet transfusions to maintain the platelet count at a safe threshold have decreased the risk of hemorrhagic complication [11,12]. However, ICH is still a frequent complication in patients with hematological malignancies, and many questions remain unanswered with regard to the clinical management of ICH in patients with hematological malignancies.

Patients with lymphoid malignancy more frequently experience central nervous system (CNS) involvement than do patients with myeloid leukemia [13,14]. ICH could be the initial presentation in patients with hematological malignancies [15,16]. The relationship of ICH and CNS involvement is still underinvestigated. Evidence on the clinical manifestations in ICH among the various hematological malignancies is relatively limited. To clarify the clinical manifestations and prognosis of ICH among patients with hematological malignancies, we retrospectively reviewed the medical records of patients admitted to the National Taiwan University Hospital between 2001 and 2010, and analyzed the data.

## Methods

### Patients and hospital setting

National Taiwan University Hospital (NTUH) is a 2,600-bed teaching hospital in northern Taiwan that provides both primary and tertiary care. We retrospectively analyzed the demographic features, hematological disease status, underlying medical diseases, laboratory and microbiological data, and outcomes of all adult patients with hematological malignancies from January 2001 to December 2010 at NTUH. The laboratory data was collected promptly after onset of ICH. We also assessed the overall 30-day mortality of these patients. This research conformed to the Helsinki Declaration and local legislation, and was approved by the National Taiwan University Hospital Research Ethics Committee.

### Chemotherapy in patients with hematological malignancies

Induction chemotherapy consisted of cytarabine and anthracycline for patients with acute myeloid leukemia (AML). Consolidation chemotherapy consisted of high-dose cytarabine-based regimens. Patients with acute promyelocytic leukemia (APL) were treated with all-trans retinoic acid (ATRA) and combined anthracycline-based chemotherapy. Patients diagnosed with acute lymphoblastic leukemia received a Cancer and Leukemia Group B 8811 (CALGB8811) [17] or Group for Research on Adult Acute Lymphoblastic Leukemia 2003 (GRAALL 2003) chemotherapy protocol [18]. Patients diagnosed with lymphoma generally received a chemotherapy regimen consisting of CHOP (cyclophosphamide, hydroxydaunorubicin (Adriamycin), vincristine (Oncovin), and prednisolone) with or without rituximab. Lymphoma patients with CNS involvement received high-dose methotrexate-based chemotherapy [19]. Patients with myeloma were treated with thalidomide, or VAD [20] (vincristine, Adriamycin, and dexamethasone) or DCEP [21] (dexamethasone, cyclophosphamide, etoposide, and cisplatin (Platin)) chemotherapy, according to the clinical decision.

### Hyperviscosity

Hyperviscosity syndrome is defined by the presence of elevated serum immunoglobulins in conjunction with clinical constitutional symptoms, bleeding, ocular, neurological, and cardiovascular manifestations [22].

### Policy of platelet transfusion in patients with hematological malignancy

The platelet counts were checked three times per week during chemotherapy in patients with hematological malignancies until the neutrophil counts recovered. The platelet counts were checked once to three times per week after neutrophil counts recovered according to clinical judgment. The complete blood counts were checked once per day in patients with freshly diagnosed acute promyelocytic leukemia until complete remission. Prophylactic platelet transfusions were used to maintain the platelet level above 20,000 cells/μl in patients receiving chemotherapy. Among patients with acute promyelocytic leukemia, active bleeding, and sepsis, the platelet counts were maintained above 50,000 cells/μl or more according to clinical judgment.

### Diagnosis of ICH and inclusion/exclusion criteria

Each patient's ICH diagnosis was determined from both clinical and neuroimaging findings. Either computed tomogram or magnetic resonance imaging was required to confirm the clinical diagnosis of ICH. Patients for whom ICH was suspected but unproven were excluded. Patients with ischemic stroke and subsequent hemorrhagic transformation were also excluded. Types and locations of intracranial hemorrhage were determined from radiology reports. According to the neuroimaging results, hemorrhages were classified into four types, namely intraparenchymal hemorrhage (IPH), subarachnoid hemorrhage (SAH), subdural hemorrhage (SDH), and epidural hemorrhage (EDH) [5].

### End points and overall survival

The starting point was the date of diagnosis of ICH. The primary end point was ICH-related death within 30 days. Survival time was the time interval from diagnosis of ICH to events such as death from any cause.

### Statistical analysis

For continuous variables, data are reported as the median with the range shown in brackets. For nominal variables, data are reported as the number of patients, unless specified otherwise. Survival curves were plotted using Kaplan-Meier analysis, and differences between curves were analyzed by log rank. All factors in the univariate analyses were also enrolled in multivariate analysis using Cox proportional-hazard testing. All statistical analyses were performed using SPSS 18.0 for Windows (SPSS, Chicago, IL, USA), and *P *values of less than 0.05 were considered statistically significant.

## Results

### ICH among patients of hematological malignancies

A total of 2,574 adult patients with hematological malignancies were admitted to National Taiwan University Hospital between 2001 and 2010. During that period, 72 episodes of ICH developed in 72 of these patients (48 men and 24 women); the median age of the ICH group was 56 years (range: 18 to 86 years). The ICH patients included 52 (6.3%) of 818 patients who had AML, 5 (2.0%) of 250 patients with acute lymphoblastic leukemia (ALL), 7 (0.7%) of 1,001 patients with lymphoma, and 8 (2.7%) of 291 patients with myeloma. Of the 214 patients who had chronic myeloid leukemia or chronic lymphoid leukemia, none developed ICH. Our results indicated an overall ICH incidence of 2.8% among adult patients with hematological malignancies. Patients with AML displayed a particularly high incidence of ICH compared with patients with other hematological malignancies (6.3% vs 1.1%; *P *= 0.001; odds ratio (OR): 5.89; 95% confidence interval (CI): 3.49 to 9.93). Except acute promyelocytic leukemia, the incidence of ICH is no different among AML French-American-British (FAB) subtypes. The clinical characteristics of ICH in patients with hematological malignancies are shown in Table 1.

**Table 1:** Clinical characteristics of intracranial hemorrhage among patients of different types of hematological malignancies

	Number of patients					
	
Subtype	AML (n = 43)	APL (n = 9)	ALL (n = 5)	Lymphoma (n = 7)	Myeloma (n = 8)	*P *value
Age:						0.094
≥60 years	19	2	1	6	4	
<60 years	24	7	4	1	4	
Gender:						0.461
Male	32	5	2	4	5	
Female	11	4	3	3	3	
Underlying hypertension:						0.428
Yes	12	3	0	3	1	
No	31	6	5	4	7	
Diabetes mellitus:						0.274
Yes	3	1	0	2	0	
No	40	8	5	5	8	
Sepsis:						0.096
Yes	16	5	5	4	4	
No	27	4	0	3	4	
Post HSCT:						0.347
Yes	8	0	0	0	1	
No	35	9	5	7	7	
Disease status:						0.409
Remission	6	2	0	0	0	
Active	37	7	5	7	8	
CNS involvement:						<0.001
Yes	0	0	0	5	1	
No	43	9	5	2	7	
Leukocyte count:						0.642
≥100,000 cells/μl	9	2	0	1	0	
<100,000 cells/μl	34	7	5	6	8	
Platelet count:						0.560
≥20,000 cells/μl	27	6	3	6	4	
<20,000 cells/μl	16	3	2	1	4	
Prothrombin time:						0.493
INR ≥1.5	10	2	1	1	1	
INR <1.5	32	7	4	6	7	
Location:						
Cerebral cortex	37	7	5	4	5	0.326
Basal ganglia	9	1	1	1	1	0.941
Brainstem	4	0	0	1	0	0.628
Cerebellum	4	2	0	1	3	0.213
Multiple	20	5	0	3	5	0.240
Type:						
Intraparenchymal hemorrhage	35	7	5	4	5	0.336
Subarachnoid hemorrhage	8	2	2	0	3	0.349
Subdural hemorrhage	11	2	1	4	4	0.307
Epidural hemorrhage	1	0	0	0	2	0.041
Intraventricular hemorrhage	10	4	1	3	2	0.618

ALL = acute lymphoblastic leukemia; AML = acute myeloid leukemia; APL = acute promyelocytic leukemia; CNS = central nervous system; HSCT = hematopoietic stem cell transplantation; INR = international normalized ratio.

Among the 72 patients with ICH, 46 (64%) had thrombocytopenia (platelets less than 20,000 cells/μl), 34 (47%) had sepsis, and 15 (21%) had prolongation of prothrombin time. Thrombocytopenia, sepsis and prolongation of prothrombin time are proposed as the potential major causes of ICH. In all, 28 patients had brain magnetic resonance angiography to survey the vascular abnormality during ICH. Neither aneurysm nor arteriovenous malformation was detected in this study. The clinical characteristics, including age, sex, hypertension, sepsis, remission status, and allogeneic stem cell transplantation were not statistically different among ICH patients with various hematological malignancies.

ICH patients who were diagnosed with lymphoma and myeloma had higher rates of CNS involvement than patients with other hematological malignancies (*P *<0.001). Two of five lymphoma patients with CNS involvement had been diagnosed with primary CNS lymphoma. One patient with diffuse large B cell lymphoma and one patient with primary CNS lymphoma developed ICH before the start of systemic chemotherapy. The other three lymphoma patients who developed ICH during lymphoma relapsed. One myeloma patient showed immature plasma cells in the cerebrospinal fluid, but no direct tumor involvement with the CNS. None of the eight myeloma patients had hyperviscosity syndrome during ICH. L-Asparaginase was used as a combined chemotherapy protocol in two ICH patients with ALL, but no statistically significant difference was found for other clinical manifestations among ICH patients.

Thrombocytopenia and prolongation of prothrombin time have been recognized as risk factors for CNS hemorrhage in patients with hematological malignancy [2,4]. In this study, we found patients with prolongation of prothrombin time had a trend towards developing SAH and significantly higher risk of death (*P *= 0.008). However, the platelet count and prothrombin time were not statistically significantly different among ICH patients with various hematological malignancies.

### Locations and types of ICH

The locations and types of ICH in patients with different types of hematological malignancies are shown in Table 1. ICH locations included the cerebral cortex (60 patients, 83%), basal ganglia (13 patients, 18%), cerebellum (10 patients, 14%), and brainstem (5 patients, 7%). A total of 33 patients (46%) had multifocal hemorrhage. Neuroimaging studies revealed that 56 patients (77%) had IPH, 22 patients (31%) had SDH, 15 patients (21%) had SAH, and 3 patients (4%) had EDH. In all, 22 patients had 2 or more types of ICH, including 11 patients with IPH and SAH, 6 patients with IPH and SDH, 1 patient with IPH and EDH, 1 patient with SDH and SAH, 1 patient with SDH and EDH, and 2 patients with IPH, SAH, and SDH. Hemorrhage ruptured into the ventricles (IVH) in 22 patients (31%). EDH occurred most frequently in patients with myeloma, and neuroimaging showed no definite focal lesion in the cranial vault.

The correlations between clinical characteristics and types of ICH are shown in Table 2. Younger age was associated with risk of IPH (*P *= 0.009), and more advanced age was associated with SDH (*P *= 0.002). Hyperleukocytosis (white blood cell count more than 100,000 cells/μl) showed a trend of being associated with IPH (*P *= 0.056). There were 12 patients with hyperleukocytosis at the time of ICH. All 12 patients had IPH (12/12, 100%). One patient had both IPH and SDH, but no patients had SAH and EDH.

**Table 2:** Clinical characteristics and types of intracranial hemorrhage

	IPH (n = 56)	*P *value	SDH (n = 22)	*P *value	SAH (n = 15)	*P *value
Gender:		0.137		0.180		0.552
Male	40		12		9	
Female	16		10		6	
Age:		0.009		0.002		>0.999
≥60 years	20		16		7	
<60 years	36		6		8	
Underlying hypertension:		0.335		0.565		0.324
Yes	13		7		2	
No	43		15		13	
Sepsis:		0.168		0.306		0.384
Yes	29		8		9	
No	27		14		6	
Remission status:		>0.999		0.421		0.669
Yes	6		1		2	
No	50		21		13	
CNS involvement:		0.609		0.361		>0.999
Yes	4		3		1	
No	52		19		14	
Leukocyte count:		0.056		0.090		0.060
≥100,000 cells/μl	12		1		0	
<100,000 cells/μl	44		21		15	
Platelet count:		>0.999		0.603		0.376
≥20,000 cells/μl	36		9		7	
<20,000 cells/μl	20		13		8	
Prothrombin time:		0.495		0.127		0.069
INR ≥1.5	43		2		6	
INR <1.5	13		20		9	

CNS = central nervous system; INR = international normalized ratio; IPH = intraparenchymal hemorrhage; SAH = subarachnoid hemorrhage; SDH = subdural hemorrhage.

### Correlated clinical manifestation and outcome in patients with ICH

In total, 25 patients (35%) died within 72 h of the onset of ICH, and 46 patients (64%) died within 30 days of ICH. Mortality was high among patients with EDH (3/3,100%) and SAH (14/15, 93%), but was relatively lower in patients with SDH (11/22, 50%). Most patients received conservative component therapy only, but 11 patients (15%) underwent craniotomy and hematoma evacuation (6 patients with IPH, 2 patients with SDH, 2 patients with both IPH and SDH, and 1 patient with SDH and EDH). Seven patients survived for 30 days (four patients with IPH, two patients with SDH, and one patient with both IPH and SDH). Five of seven survivors had long-term survival (more than 1 year) with minimal neurological sequelae. Surgical intervention showed a trend of improved 30-day outcome (*P *= 0.085).

To clarify the clinical manifestations for the 30-day outcome in patients with hematological malignancies, we analyzed clinical prognostic factors as shown in Table 3. Univariate analysis revealed that sepsis (*P *= 0.05), SAH (*P *= 0.007), multifocal cerebral hemorrhage (*P *= 0.006), and prolonged prothrombin duration (*P *= 0.013) were associated with poor prognosis. The type of hematological malignancy had no effect on the 30-day outcome, nor did allogeneic stem cell transplantation.

**Table 3:** Univariate analysis of 30-day outcome of ICH among patients with hematological malignancies.

	Alive (n = 26)	Dead (n = 46)	Univariate analysis, *P *value	Multivariate analysis, *P *value (odds ratio; 95% CI)
Gender:			0.604	
Male	16	32		
Female	10	14		
Age			>0.999	
≥60 years	12	20		
<60 years	14	26		
Hematological malignancies			0.679	
AML	14	29		
APL	5	4		
ALL	2	3		
Lymphoma	3	4		
Myeloma	2	6		
Remission status			0.448	
Yes	4	4		
No	22	42		
Post HSCT			0.473	
Yes	2	7		
No	24	39		
Underlying hypertension			>0.999	
Yes	7	12		
No	19	34		
Sepsis			0.050	
Yes	8	26		
No	18	20		
CNS involvement			>0.999	
Yes	2	4		
No	24	42		
Subarachnoid hemorrhage			0.007	0.021 (10.9; 1.3 to 88.9)
Yes	1	14		
No	25	32		
Multifocal hemorrhage			0.006	0.026 (4.7; 1.6 to 14.0)
Yes	6	27		
No	20	19		
Surgical intervention			0.085	
Yes	7	4		
No	19	41		
Leukocyte count			>0.999	
≥100,000 cells/μl	4	8		
<100,000 cells/μl	22	38		
Platelet count			0.802	
≥20,000 cells/μl	16	30		
<20,000 cells/μl	10	16		
Prolongation of prothrombin time			0.013	0.008 (10.9; 1.3 to 85.5)
INR ≥1.5	1	14		
INR <1.5	24	32		

ALL = acute lymphoblastic leukemia; AML = acute myeloid leukemia; APL = acute promyelocytic leukemia; CNS = central nervous system; HSCT = hematopoietic stem cell transplantation; ICH = intracranial hemorrhage; INR = international normalized ratio.

All covariates with a *P *value less than 0.1 were enrolled in the multivariate analysis. Multivariate analysis was conducted using the Cox proportional hazard test, shown in Table 3. Poor outcome of ICH in patients with hematological malignancies was associated with three independent factors, namely prolonged prothrombin duration (*P *= 0.008; OR 10.5; 95% CI 1.3 to 85.5), SAH (*P *= 0.021; OR 10.9; 95% CI 1.3 to 89.9), and multifocal cerebral hemorrhage (*P *= 0.026; OR 4.7; 95% CI 1.6 to 14.0). Surgical intervention showed a trend of being associated with improved prognosis in the univariate analysis (*P *= 0.085), but no statistical significance was found for this relationship in the multivariate analysis.

## Discussion

The cause of ICH is very important and holds implications for further therapy. Since no patient received autopsy after ICH, the definite cause of ICH in patients with hematological malignancies is inconclusive. Among the 72 patients with ICH, 46 (64%) patients had thrombocytopenia (platelets less than 20,000 cells/μl), 34 (47%) had sepsis, and 15 (21%) had prolongation of prothrombin time. Consequently, thrombocytopenia, sepsis and prolongation of prothrombin time are the major predisposing factors for ICH.

The definite causes of ICH could be further investigated by autopsy study. Occurrence of ICH in patients with acute leukemia has been reported [3,4]. Case reports of ICH included patients with primary CNS lymphoma [23-25], intravascular lymphoma [26,27], and myeloma [28,29]. However, comparisons of the clinical characteristics and outcome in patients with various hematological malignancies were limited. The current study reveals that patients with AML had a higher incidence of ICH than patients with other hematological malignancies (6.3% vs 1.1%; *P *= 0.001).

The incidence of ICH is less common in patients with lymphoma and myeloma, among those with intracranial involvement. ICH occurred more frequently in patients with CNS involved lymphoid malignancy relative to those with CNS involved acute leukemia. The pathogenesis of ICH among patients with various hematological malignancies may be different. Leukemias are circulating precursors of lymphoblastic or myeloid neoplasm that involve the bone marrow and peripheral blood. Leukemias often exert an indirect effect on the nervous system, causing thrombocytopenia, coagulation factor deficiency, sepsis, therapy-related complications, and vessel wall abnormalities [9]. In contrast, for most lymphoma and myeloma patients, the tumor cells do not invade tissues and vessels. As Glass [9] proposed, the tumor cells may exert direct effects as solid tissue tumors on cerebral tissue and blood vessels, resulting in vascular occlusion and end-organ ischemia, which in turn, potentially cause thrombosis and hemorrhagic transformation. In the current study, we considered that ICH might partly contribute to the direct invasion or intravascular involvement of tumor cells in patients with lymphoma and myeloma, and the definite mechanism of lymphoma and myeloma associated ICH should be further investigated.

Patients with CNS lymphoma or myeloma involvement should be aware of the increased risk of ICH [23-27]. L-Asparaginase is one of the combined chemotherapy agents frequently used to treat acute lymphoblastic leukemia; it may decrease the plasma antithrombin and fibrinogen levels, potentially inducing bleeding [30]. Hyperviscosity syndrome and direct tumor invasion also have been reported, which could lead to fatal ICH in patients with multiple myelomas [28,29]. L-asparaginase and myeloma associated hyperviscosity syndrome implicate bleeding tendency, however, both factors did not disclose the clinical significance of outcome in ICH patients with hematological malignancies in the current study

The types and locations of ICH events in patients with hematological malignancies are also rarely reported [2,5,31]. A rapid onset of focal neurological deficit with clinical signs of increased intracranial pressure is strongly suggestive of a diagnosis of ICH, but cranial imaging is required to differentiate it from ischemic stroke [32,33]. In this study, neuroimaging not only disclosed the types and locations of ICH, but could also predict the outcome of patients with ICH. A neuroimaging study should be performed rapidly after symptoms or signs of neurological deficit in patients with hematological malignancies.

We found most (78%) ICH events in patients with hematological malignancies tended to be IPH, which was congruent with prior reports [5,34]. Subdural hemorrhage is common among older members of the general population [35]. Among our study patients with hematological malignancies, older patients had more SDH than younger patients (*P *= 0.002). Symptoms of SDH have a slower onset than those of ICH, because the lower pressure in veins causes them to bleed more slowly than arteries [35]. Given that SDH is associated with a relatively good prognosis in patients with hematological malignancies, clinical awareness of neurological symptoms and signs in older patients, and the rapid use of neuroimaging, could improve diagnostic accuracy and thus the outcome of ICH. Although SAH represents only 5% of all strokes, it is responsible for 25% of all fatalities related to stroke in the general population [36].

In the current study, SAH affected only 21% patients with hematological malignancies but the mortality rate in the SAH group was 90%. Advances have been made in surgical intervention for SAH [36], but none of the SAH patients in the current study received surgery. Surgical intervention showed a trend of improved 30-day outcome (*P *= 0.085) in the current study. Further research or surgical intervention is needed to improve the prognosis for SAH patients with hematological malignancies. Hemorrhage extending into the ventricles is associated with obstructive hydrocephalus and a relatively poor prognosis in ICH of general population [29,37]. Most patients with hematological malignancies who developed ICH had a poor prognosis. IVH is not an independent poor prognostic factor in patients with hematological malignancies.

Multivariate analysis using Cox's proportional hazard test revealed that ICH in patients with hematological malignancies was associated with three independent factors. These were prolonged prothrombin duration (*P *= 0.008; OR 10.5; 95% CI 1.3 to 85.5), subarachnoid hemorrhage (*P *= 0.021; OR 10.9; 95% CI 1.3 to 89.9), and multifocal cerebral hemorrhage (*P *= 0.026; OR 4.7; 95% CI 1.6 to 14.0). Several studies have demonstrated that prolongation of prothrombin duration is related to fatal ICH outcome in patients with hematological malignancies [2-4]. The current study examined prolonged prothrombin duration as one of the independent prognostic factors for ICH in patients with hematological malignancies. Leukemic cells may express fibrinolysis and other proteolysis enzymes, which has been implicated in the pathogenesis of bleeding in some patients [38-40]. Chemotherapy-related endothelial injury and reduction of coagulation factors also could play a role in the pathogenesis [38,39]. Coagulopathy in patients with hematological malignancies could play an important role in the pathogenesis of ICH. Prolongation of prothrombin time could be ignored during the period of chemotherapy of patients with hematological malignancies; we suggest regular follow-up of prothrombin time and prompt correction in patients with hematological malignancies. This could potentially decrease the mortality of ICH .

A total of 11 patients underwent craniotomy and hematoma evacuation. Surgical intervention showed a trend of improved 30-day outcome (*P *= 0.085) in ICH patients with hematological malignancies. Five of the seven survivors had long-term survival (more than 1 year) with minimal neurological sequelae. Although few patients underwent surgical intervention, we would advocate aggressive surgical treatment such as craniotomy and hematoma evacuation for those patients with non-refractory hematological malignancies. Recently, activated factor VII has been used successfully in patients with ICH [40-42]. A subsequent phase III clinical trial revealed that hemostatic therapy with rFVIIa reduced the growth of hematomas, but did not improve patient survival or functional outcome after intracerebral hemorrhage [43]. Further clinical trials should be conducted in ICH patients with hematological malignancies.

There were some limitations to this study. First, we tried to investigate all the adult patients with hematological malignancies. However, this study is a retrospective cohort in a single university hospital between 2001 and 2010. Given the secular changes in the rapidly evolving field of hematology, patients may have received different chemotherapy protocols and supportive management. Second, thrombocytopenia (64%), sepsis (47%) and prolongation of prothrombin time (21%) were the most common risk factors of ICH. However, there was no patient autopsied for the definite cause of ICH. Furthermore, a prospective multicenter clinical study should be conducted to define the incidence of ICH in this population. We limited the definition of cases to patients with positive neuroimaging study to prevent confounding factors. Consequently, a few patients with refractory leukemia and lymphoma who did not consent to neuroimaging studies were excluded from this study.

## Conclusions

ICH is more common in patients with AML than in patients with other types of hematological malignancy. ICH occurred in patients with lymphoma when there was CNS neoplastic involvement. Prognosis did not differ according to type of hematological malignancy. Prolonged prothrombin duration, SAH, and multiple-site hemorrhage were all independent prognostic factors of ICH in patients with hematological malignancies. Neuroimaging studies of the location and type of ICH could assist with outcome prediction for patients with hematological malignancies.

## Competing interests

The authors declare they have no competing interests.

## Authors' contributions

C-YC, C-HT, H-CW, and J-HL participated in the study design. S-YH, WT, MY, P-YC, J-LT and H-FT participated in data collection and analysis. C-YC, C-HT, AC, and H-FT participated in editing and proofreading. All authors read and approved the final manuscript.

## Pre-publication history

The pre-publication history for this paper can be accessed here:

http://www.biomedcentral.com/1741-7015/10/97/prepub
